# Metoclopramide-induced Serotonin Syndrome

**DOI:** 10.7759/cureus.6359

**Published:** 2019-12-12

**Authors:** Sreenath Meegada, Rajiv Prakash Heda, Sanjaya Satapathy, Rajanshu Verma

**Affiliations:** 1 Internal Medicine, The University of Texas Health Science Center/Christus Good Shepherd Medical Center, Longview, USA; 2 Internal Medicine, University of Tennessee Health Science Center, Memphis, USA; 3 Sandra Atlas Bass Center for Liver Diseases & Transplantation, North Shore University Hospital/Northwell Health, Manhasset, USA; 4 Gastroenterology, University of Tennessee Health Science Center, Memphis, USA

**Keywords:** metoclopramide, serotonin syndrome, selective serotonin reuptake inhibitors (ssri)

## Abstract

Serotonin syndrome is a clinical diagnosis characterized by a constellation of autonomic and neurological physical examination findings due to the use of one or more serotonergic agents. Due to high morbidity and mortality associated with this condition, high index of suspicion is required in making this diagnosis. Treatment is aimed at discontinuation of the offending agent and supportive care. We present a case of a 28-year-old woman who presented with acetaminophen toxicity, however developed iatrogenic serotonin syndrome due to use of scheduled intravenous metoclopramide. Metoclopramide, by itself, very rarely causes serotonin syndrome and typically results in this condition when used in combination with other pro-serotonergic agents.

## Introduction

Serotonin syndrome is a life-threatening condition resulting from serotonergic excess from therapeutic drug use, drug overdose or drug-drug interactions [1]. Various drugs have been implicated in causation of serotonin syndrome such as selective serotonin reuptake inhibitors (SSRIs), tricyclic antidepressants, monoamine oxidase inhibitors (MAOIs), opiate analgesics, over the counter cough medicines, antibiotics, anti-emetics, anti-migraine agents, and herbal products [1]. The common clinical presentation is altered mental status, restlessness, myoclonus, hyperreflexia, shivering, tremor, and diaphoresis [2]. Diagnosing this entity can be difficult due to its dependence on taking a good medication history and physical examination findings alone, but has been made easier with the use of Hunter Serotonin Toxicity Criteria which have a high sensitivity and specificity [3]. Treatment of the serotonin syndrome involves stopping the offending drug/drugs, controlling body temperature, agitation, and autonomic instability, administering serotonin antagonists and providing supportive care [4]. This case illustrates the importance of recognizing metoclopramide as a rare but significant cause of precipitating serotonin syndrome.

## Case presentation

A 28-year-old Caucasian woman presented to the emergency department (ED) with complaints of nausea, vomiting, abdominal pain and headache after ingesting a “hand full” (approximately 50) of 500 mg acetaminophen tablets as a suicide attempt gesture four days ago after having an altercation with her mother. Past medical history was significant for depression, anxiety, previous suicide attempts (with pill ingestions and wrist slitting), sexual abuse (rape), benzodiazepine addiction (underwent rehabilitation) and migraines. Her home prescription medications included buspirone and paroxetine. She had used amitriptyline in the past as well. She denied any history of drinking alcohol, smoking cigarettes or using illicit drugs. There was no family history of psychiatric disorders or liver-related diseases.

Vital signs showed a blood pressure of 111/59 mm Hg, temperature of 98.8 degrees Fahrenheit, heart rate 139 beats/min, respiratory rate 18 breaths/min, and oxygen saturation 97% on room air. Physical examination showed an alert yet anxious patient who had no signs of jaundice, bruising, hepatic encephalopathy or asterixis. Neurological exam on initial presentation revealed normal reflexes. On abdominal exam, there was tenderness to palpation in the right upper quadrant with normal active bowel sounds.

Investigations

**Figure 1:**
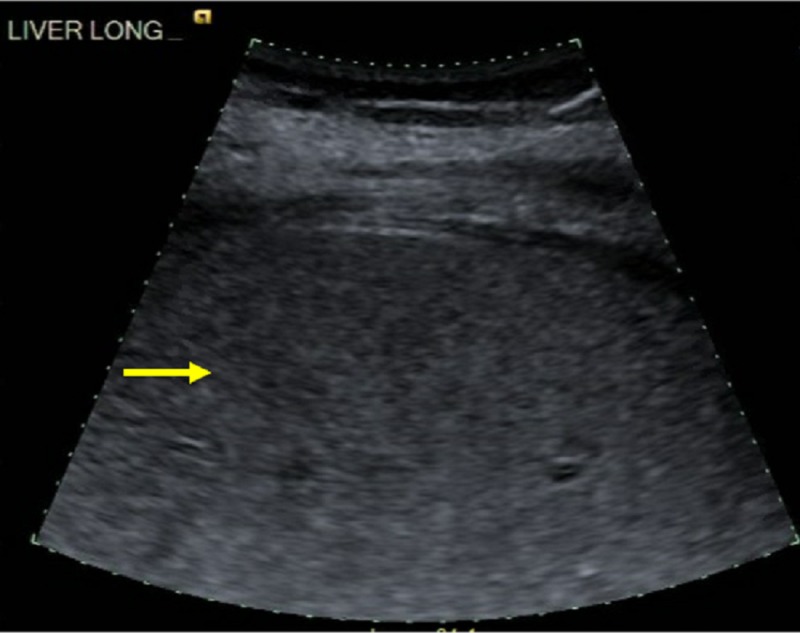
Ultrasound abdomen liver long axis showing steatosis

Serum hematology tests showed white blood cell count 15,500 cells/micro liter (normal: 4,200-10,200), hemoglobin 10.8 (normal: 11.5-14.8), platelets 99,000 cells/micro liter (normal: 150,000-400,000), International Normalized Ratio (INR) 5.3 (normal: 0.8-1.0), prothrombin time (PT) 47.9 sec (normal: 11.7-14.5). Serum chemistry tests showed sodium 138 mEq/L (normal: 136-145), potassium 5.2 mEq/L (normal: 3.5-5.1), chloride 102 mEq/L (normal: 98-107), bicarbonate 15 mEq/L (normal: 22-32), anion gap 25 mEq/L after correcting for albumin (normal: 8-12), blood urea nitrogen 37 mg/dL (normal: 7-18), creatinine 3.31 mg/dL (normal: 0.52-1.21), estimated glomerular filtration rate (eGFR) 16.6 mL/min/1.73 m^2^ (normal: > 60), albumin 2.6 g/dl (normal: 3.4-5.0), total bilirubin 3.4 mg/dl (normal: 0.2-1.0), alkaline phosphatase 142 U/L (normal: 45-117), AST 9368 U/L (normal: 15-37), ALT 7982 U/L (normal: 13-56), lactic acid 11.1 mEq/L (normal: 0.4-2.0), acetaminophen level 8.8 mcg/mL. Urine drug screen for illicit substances was negative. Ultrasound of the abdomen done in the ED showed hepatic steatosis (see Figure 1) but no biliary dilatation.

The patient was diagnosed with high anion gap metabolic acidosis/lactic acidosis, acute kidney injury, elevated INR due to acetaminophen toxicity and was started on an intravenous N-acetylcysteine drip after initial volume resuscitation. She received intravenous vitamin K which helped correct her INR to 2.8. Given elevated transaminases, all her home prescription medications (including bupropion and paroxetine) were held on admission. In order to address her persistent nausea and vomiting, she was also started on scheduled intravenous metoclopramide 10 mg every six hours the following day by the hospital medicine team. With resuscitation and conservative management, her condition continued to improve during five days of her hospital stay with improvement in transaminases (AST 98 and ALT 851 U/L), acidosis/lactic acid (1.3 mmol/L), INR (1.5) and creatinine (1.4 mg/dl) by the time of discharge. Intravenous N-acetylcysteine was discontinued on hospital day 5. However, as a result of scheduled every six-hour intravenous metoclopramide use, on hospital day 4, she was found to have a blood pressure of 146/109 mmHg, heart rate 120 beats/min, clonus, tremors and hyperreflexia (see Video 1). A diagnosis of metoclopramide-induced serotonin syndrome was made. Metoclopramide was promptly discontinued. Given her history of benzodiazepine addiction, patient and family members were reluctant to try intravenous lorazepam for symptom mitigation. Serotonin syndrome promptly resolved over the next 36 hours and she was discharged to a mental health facility under the care of a psychiatrist. The patient was seen in outpatient clinic two weeks later when her liver enzymes, creatinine, INR had normalized and the patient was back to her usual state of health.

**Video 1:** Metoclopramide-induced serotonin syndrome

## Discussion

Serotonin syndrome is a clinical diagnosis which results from increased serotonergic activity characterized by mental status changes, neuromuscular irritability and autonomic lability in the setting of intake of one or more pro-serotonergic agents. Serotonin (5-HT) is a neurotransmitter present in the central nervous system where it plays a role in regulating cognition, mood, attention span and temperature control. In peripheral nervous system, serotonin is produced by the enterochromaffin cells in the gastrointestinal tract and regulates gastrointestinal motility which explains the emesis and diarrhea seen with serotonin syndrome. Serotonin also plays a role in platelet aggregation, bronchial, uterine and vasoconstriction. Symptoms associated with serotonin syndrome include diarrhea, high blood pressure, nausea, vomiting, tachycardia, hyperthermia, diaphoresis, mydriasis, agitation, delirium, disorientation, muscle rigidity, tremor, hyperreflexia, and clonus. Symptoms of hyperreflexia, rigidity and clonus are more pronounced in the lower extremities [1].

Establishing the clinical diagnosis of serotonin syndrome is through the Hunter Toxicity Criteria Decision Rules, otherwise known as the Hunter Criteria which were published in 2003 [3]. In order to meet the Hunter Criteria, patient must be on at least one serotonergic medication and meet one of the following physical examination findings: spontaneous clonus, inducible clonus or ocular clonus with agitation/diaphoresis, tremor with hyperreflexia, or hypertonic muscle tone with temperature greater than 38 degrees centigrade with ocular or inducible clonus [3].

Serotonin syndrome may be caused by therapeutic misadventure, drug-drug interactions, intentional drug overdose or illicit drug use [1]. Several classes of serotonergic agents have been implicated in the causation of serotonin syndrome. These include SSRI, serotonin-norepinephrine reuptake inhibitors (SNRI), dopamine-norepinephrine reuptake inhibitors (DNRI), tricyclic antidepressants (TCAs), MAOI, dopamine precursors, 5-HT3 receptor blockers, ergot derivatives, triptans, amphetamine derivatives, lithium and illicit drugs, e.g., cocaine, ecstasy (MDMA) and lysergic acid diethylamide (LSD) [1]. Other miscellaneous drugs reported to contribute to the occurrence of serotonin syndrome include linezolid, ritonavir, valproate, carbamazepine, tramadol, sibutramine, St. John’s wort, buspirone, L-tryptophan, ginseng, dextromethorphan, cyclobenzaprine, methylene blue and metoclopramide [1, 5, 6].

Metoclopramide is a prokinetic agent used as an anti-emetic for the treatment of chemotherapy-induced or post-operative nausea and vomiting, diabetic gastroparesis, gastro-esophageal reflux disease, small bowel obstruction and radiologic examination of upper gastrointestinal tract [7]. Metoclopramide has multiple mechanisms of action such as antagonism/inhibition of pre- and post-synaptic dopamine-2 (D2) receptors in the gut wall, stimulation of presynaptic 5-HT4 receptors and releasing acetylcholine from cholinergic neurons, inhibition of D2 and 5-HT3 receptors in the chemoreceptor trigger zone [8]. Metoclopramide contributes to serotonin syndrome by impairing its reuptake from the synaptic cleft into the presynaptic neuron [9]. Metoclopramide is metabolized through the liver and excreted in urine. Thus, patients with liver and/or renal failure have impaired clearance of its drug metabolites and patients with renal failure should receive 50% of the normal dose [10]. Side effects of metoclopramide include hyperprolactinemia/galactorrhea, amenorrhea, gynecomastia, agranulocytosis/neutropenia, angio-edema, hepatotoxicity, seizures, hallucinations, depression, suicidality, methemoglobinemia, atrioventricular block, acute congestive heart failure and others. It also has extrapyramidal side effects such as parkinsonism, dystonia, tardive dyskinesia and neuroleptic malignant syndrome [11]. Serotonin syndrome due to metoclopramide has been reported in isolated cases in which patients were concomitantly taking either mirtazapine, linezolid, olanzapine and fluoxetine, lithium and imipramine or other selective serotonin reuptake inhibitors [8, 12-14]. To the best of our knowledge, there are currently no reported cases of serotonin syndrome caused by metoclopramide alone.

Treatment of serotonin syndrome revolves around stopping the offending agents, providing symptomatic and supportive care and sedation with benzodiazepines or treatment with serotonin antagonist, cyproheptadine if necessary. Intravenous fluids, anti-pyretic measures are used to address volume depletion, hyperthermia and benzodiazepines are used to control the agitation. Serotonin antagonism is achieved through inhibition of the 5-HT1A and 5-HT2A receptor with cyproheptadine. If the serotonin syndrome is caused by an intentional overdose, it is important to also check serum acetaminophen, salicylate, alcohol levels and a urine drug screen [15].

Based on our extensive review of medical literature, this is the first case of serotonin syndrome induced by metoclopramide alone. Although both buspirone (half-life three hours) and paroxetine (half-life 21 hours) were held four days prior to the development of serotonin syndrome, given presence of renal failure, possibility of extended retention of SSRI metabolites contributing to serotonin syndrome cannot be completely ruled out. Through the medium of this article, we want to emphasize upon our readers that in addition to the irreversible side effect of tardive dyskinesia, scheduled high dose metoclopramide is associated with the development of serotonin syndrome as well and that it should be used sparingly for the treatment of nausea and vomiting.

## Conclusions

Serotonin syndrome is a clinical diagnosis made on the basis of mental status changes, autonomic instability and neuromuscular excitability in the presence of a serotonergic agent. It is an easily missed diagnosis and so high index of suspicion is required in patients who are on one or more serotonergic agents. Metoclopramide is a rare though important (given its frequent use) contributor to serotonin syndrome. Patients with impaired hepatic metabolism and renal excretion may retain serotonergic metabolites for an extended period of time, thus increasing the risk of development of serotonin syndrome. Treatment of serotonin syndrome involves discontinuing the offending agent, providing hemodynamic support, using benzodiazepines for sedation and cyproheptadine to antagonize overt serotonergic activity.
